# Assessment of RNAi-induced silencing in banana (*Musa* spp.)

**DOI:** 10.1186/1756-0500-7-655

**Published:** 2014-09-18

**Authors:** Tuong Vi T Dang, Saskia Windelinckx, Isabelle M Henry, Barbara De Coninck, Bruno PA Cammue, Rony Swennen, Serge Remy

**Affiliations:** Laboratory of Tropical Crop Improvement, Department of Biosystems, KU Leuven, Willem de Croylaan 42, 3001 Leuven, Belgium; Department of Plant Biology and Genome Center, U.C.Davis, 451 E. Health Sciences Drive, Davis, CA 95616 USA; Center of Microbial and Plant Genetics, Department of Microbial and Molecular Systems, KU Leuven, Kasteelpark Arenberg 20, 3001 Leuven, Belgium; Bioversity International, Willem de Croylaan 42 bus 2455, 3001 Leuven, Belgium; International Institute of Tropical Agriculture, P.O. Box 10, Duluti, Arusha, Tanzania; Department of Plant Systems Biology, VIB, Technologiepark 927, 9052 Ghent, Belgium

**Keywords:** Banana, Embryogenic cell suspension, GUS expression, ihpRNA vector, Transgene silencing

## Abstract

**Background:**

In plants, RNA- based gene silencing mediated by small RNAs functions at the transcriptional or post-transcriptional level to negatively regulate target genes, repetitive sequences, viral RNAs and/or transposon elements. Post-transcriptional gene silencing (PTGS) or the RNA interference (RNAi) approach has been achieved in a wide range of plant species for inhibiting the expression of target genes by generating double-stranded RNA (dsRNA). However, to our knowledge, successful RNAi-application to knock-down endogenous genes has not been reported in the important staple food crop banana.

**Results:**

Using embryogenic cell suspension (ECS) transformed with ß-glucuronidase (GUS) as a model system, we assessed silencing of *gusA*^*INT*^ using three intron-spliced hairpin RNA (ihpRNA) constructs containing *gusA*^*INT*^ sequences of 299-nt, 26-nt and 19-nt, respectively. Their silencing potential was analysed in 2 different experimental set-ups. In the first, *Agrobacterium*-mediated co-transformation of banana ECS with a *gusA*^*INT*^ containing vector and an ihpRNA construct resulted in a significantly reduced GUS enzyme activity 6–8 days after co-cultivation with either the 299-nt and 19-nt ihpRNA vectors. In the second approach, these ihpRNA constructs were transferred to stable GUS-expressing ECS and their silencing potential was evaluated in the regenerated *in vitro* plants. In comparison to control plants, transgenic plants transformed with the 299-nt *gusA*^*INT*^ targeting sequence showed a 4.5 fold down-regulated *gusA* mRNA expression level, while GUS enzyme activity was reduced by 9 fold. Histochemical staining of plant tissues confirmed these findings. Northern blotting used to detect the expression of siRNA in the 299-nt ihpRNA vector transgenic *in vitro* plants revealed a negative relationship between siRNA expression and GUS enzyme activity. In contrast, no reduction in GUS activity or GUS mRNA expression occurred in the regenerated lines transformed with either of the two *gusA*^*INT*^ oligo target sequences (26-nt and 19-nt).

**Conclusions:**

RNAi-induced silencing was achieved in banana, both at transient and stable level, resulting in significant reduction of gene expression and enzyme activity. The success of silencing was dependent on the targeted region of the target gene. The successful generation of transgenic ECS for second transformation with (an)other construct(s) can be of value for functional genomics research in banana.

**Electronic supplementary material:**

The online version of this article (doi:10.1186/1756-0500-7-655) contains supplementary material, which is available to authorized users.

## Background

Agronomically important genes are cloned for genetic improvement in crop to generate transgenic plants superior to existing varieties in terms of disease and pest resistance, drought tolerance, yield, vigour, etc. The newly sequenced genome of DH-Pahang, a homozygous doubled-haploid *Musa acuminata* (2*n* = 22), and of *Musa balbisiana*, a heterozygous genotype (2*n* = 22) will greatly facilitate this type of functional genomics studies in banana (*Musa* spp.) [1, 2]. To determine gene function, a common strategy is to generate lines that express no or reduced activity of one or more genes via insertional mutagenesis or RNAi-induced gene silencing, and subsequent study of the phenotypes of the knock-out or knock-down lines [3]. Complementary to this approach, the function of a gene is also investigated by over-expression [4], a technique already routinely applicable to banana [5–7]. Insertional mutagenesis for down-regulation, based on transposons or T-DNA integration has been achieved in various plant species including Arabidopsis (*Arabidopsis thaliana*) [8], maize (*Zea mays*) [9], rice (*Oryza sativa*) [10], *Medicago truncatula* [11] and tomato (*Lycopersicon esculentum*) [12]. We have successfully applied insertional mutagenesis to trap functional banana promoters [13, 14]. Nonetheless, insertional mutagenesis is hampered by complex T-DNA insertions which might cause DNA rearrangement, lethal knock-outs, or the necessity of making homozygous lines which is impossible for edible bananas due to their sterility. In addition, this is an inherently random approach limited by (i) its non-sequence-specific gene silencing activity, (ii) gene redundancy often present in gene families, and (iii) polyploidy in the case of for example, wheat and banana. The RNAi-induced gene silencing approach can overcome these disadvantages since it mediates PTGS of the target gene(s), resulting in inhibition of gene expression in a sequence-specific manner via the formation of double-stranded RNA (dsRNA) [15, 16]. Consequently, it allows silencing of single or multiple members of a gene family or, of homologous gene copies in polyploids by targeting unique sequences shared by these gene members [17–19].

RNAi is an evolutionarily conserved mechanism for gene silencing in eukaryotes in which dsRNA functions as a trigger directing homology-dependent silencing of the target gene(s) [20, 21]. The RNAi silencing pathway in plants starts with the formation of long dsRNA precursors [22]. Distinct DICER-like enzymes recognize and then cleave the long dsRNA molecules into 21-, 22- and 24-nt short interference RNAs (siRNAs) [22–25]. The 21-nt siRNAs mediate post-transcriptional gene silencing (PTGS), whereas the 24-nt siRNAs guide RNA-directed DNA methylation (RdDM) of homologous DNA (i.e., transcriptional gene silencing). The 22-nt siRNAs are thought to act as backup for both 21-nt and 24-nt siRNAs [22, 26]. One strand of the siRNA duplex is loaded on one of the AGO (Argonaute) proteins to form the core of the RNA-induced silencing complex (RISC). The siRNA-guided AGO proteins mediate degradation of target mRNAs or DNA methylation [27].

The RNAi-induced gene silencing approach is utilized to generate loss-of-function mutants (knock-down or knock-out) and its high efficiency, specificity and easiness render it useful for genome-wide analysis of gene function [3, 28]. RNAi was proven efficient in stable gene silencing in various monocot crop plants such as maize (*Zea mays*) [29], wheat (*Triticum aestivum*) [19], rice (*Oryza sativa*) [18, 30, 31], sugarcane (*Saccharum* spp. Hybrid) [32], and barley (*Hordeum vulgare*) [33]. However, to the best of our knowledge no study on the use of RNAi to silence endogenous genes in banana exists.

Assessment of RNAi-induced gene silencing usually involves the silencing of a native gene resulting in a visibly screenable phenotype. For example, RNAi-mediated silencing of the *phytoene desaturase* (*PDS*) gene in the diploid *A. thaliana* [34] and the polyploid *Triticum aestivum* [19] resulted in readily visible photobleached leaves; or silencing of *CHS* gene family in apple (*Malus x domestica*) by RNAi caused clearly reduced anthocyanins and abnormal plant phenotypes [35]. However, such approach presumes the prior isolation of the endogenous genes (e.g. *PDS* or *CHS*) which was not feasible in banana because its genome was not yet released at the start of this study. Alternatively, we opted for β-glucuronidase (GUS) encoded by the *gusA* reporter gene for assessment of RNAi-induced gene silencing in banana. Besides precise quantification of the level of silencing, the use of a reporter gene system allows measurements at the mRNA abundance and enzyme activity levels. The relationship between these two measurements reveals to what extent gene silencing pushes through to the level that determines the phenotype.

This study encompassed two consecutive phases. First, a transient co-transformation approach was applied in which an intron-spliced *gusA* gene (*gusA*^*INT*^) vector and different *gusA*^*INT*^ targeting ihpRNA vectors were co-introduced into banana embryogenic cell suspensions (ECS) for an early assessment of the feasibility of gene silencing. Encouraging results were obtained with two ihpRNA vectors. Next, to better mimic native gene silencing, a stable GUS-expressing ECS was first generated and independently transformed with each of the ihpRNA vectors. The level of silencing was monitored at different stages during regeneration of transgenic *in vitro* lines. The ihpRNA vector carrying a 299-nt *gusA*^*INT*^ targeting sequence effectively reduced *gusA*^*INT*^ expression at all developmental stages and in different plant tissues.

## Results

### The transient GUS silencing system yields promising siRNA-mediated silencing results

#### ihpRNA vectors construction

**Figure 1:**
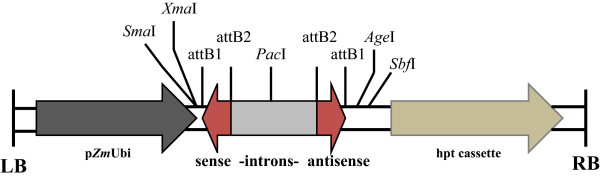
**Binary T-DNA vector backbones.** Schematic presentation of the T-DNA region of the three ihpRNA vectors, pIMHKUL3, pIMHKUL4 and pIMHKUL5 containing 299-nt, 26-nt and 19-nt RNA duplexes, respectively. LB, T-DNA left border; pZmUbi, *Zea mays* polyubiquitin promoter; sense-introns-antisense, 299-nt, 26-nt or 19-nt inverted repeats (red) separated by castor bean catalase (cat) and pyruvate dehydrogenase kinase (Pdk) introns (grey); hpt cassette containing CaMV 35S promoter, hygromycine resistance gene and NOS terminator; RB, T-DNA right border.

To assess RNAi-induced silencing of *gusA*^*INT*^ at the transient level, we constructed three ihpRNA constructs pIMHKUL3, pIMHKUL4 and pIMHKUL5 containing 299-nt, 26-nt, and 19-nt of the *gusA* genomic sequence, respectively, and targeting different sites of the *gusA* mRNA (see Methods, section ‘Preparation of RNAi vectors’). Each vector harboured a sense and antisense *gusA* sequence linked through a spacer and under control of the *Zea mais Ubi* promoter (Figure 1). Construction of the 19-nt *gusA* sequence was based on the report by Lu et al. [36] demonstrating that ihpRNA constructs containing this sequence were able to efficiently suppress *gusA* expression in GUS expressing tobacco (*Nicotiana benthamiana*) plants. A target sequence of a few hundred base pairs is known to be functional for gene silencing [19, 37]. Therefore, a 299-nt *gusA* sequence more near to the 3′ end of the *gusA* gene was cloned in the same backbone vector yielding pIMHKUL3. Finally, because we have identified more than 40,000 SuperSAGE tags previously [38] Remy et al., unpublished results], representing native banana genes of unknown functions, we wanted to test whether the 26-nt 3′ end SuperSAGE tag sequence could also be exploited to silence a (trans)gene. Hence, the 26 bp downstream of the last *Nla*III restriction site (CATG) of the *gusA* gene sequence was used for the construction of the pIMHKUL4 vector (Additional file 1).

#### Transient GUS silencing assay

**Figure 2:**
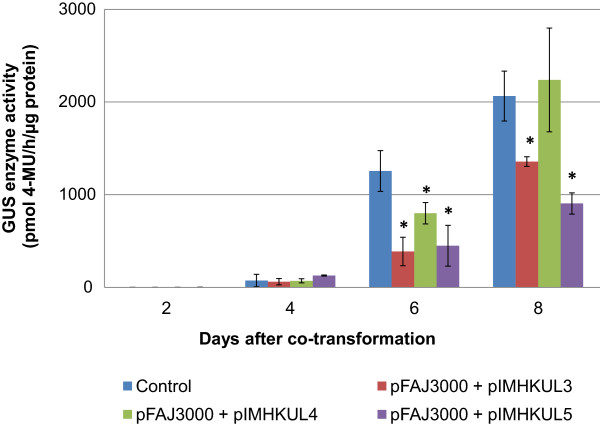
**Transient GUS enzyme activity following co-transformation.** Transient GUS enzyme activity in ECS samples from 2 to 8 days after co-transformation of pFAJ3000 together with pSTARGATE (control) or with one of the ihpRNA vectors. Bars represent the mean ± SD. Mean values were calculated from six independent biological replicates (n = 6). Significant differences between each of the treatments and the control were analyzed using one-way ANOVA (*, P < 0.05).

Our goal was to be able to easily and relatively quickly assess the silencing capabilities of the constructed ihpRNA vectors in banana. Therefore, a transient GUS silencing system was established by *Agrobacterium*-mediated co-transforming each of the ihpRNA vectors together with *gusA*^*INT*^ containing vector (pFAJ3000) into an untransformed banana ECS [39]. Co-transformation of the cloning vector pSTARGATE and pFAJ3000 was included as a control. At days 2 and 4 after co-transformation, no significant difference in GUS activity was detected between this control treatment and any of the co-transformed ihpRNA vectors (Figure 2). By contrast, at day 6 after co-transformation, transient GUS activity of all three co-transformed ihpRNA vectors was significantly lower than that of the control. In the pIMHKUL3 and pIMHKUL5 co-transformed samples, GUS activity was almost 3-fold lower compared with the control (Figure 2). The suppression of GUS activity in the pIMHKUL4 co-transformed samples disappeared at day 8 after co-transformation, whereas pIMHKUL3 and pIMHKUL5 continued to exert a significant reduction on GUS activity compared to the control. In conclusion, these results showed that vectors pIMHKUL3 and pIMHKUL5 containing a 299-nt and 19-nt *gusA* target sequence, respectively, are able to strongly inhibit transient GUS expression and that pIMHKUL4, containing the SAGE tag was able to do so less efficiently.

### Silencing of stable GUS expression

#### Generation of stable GUS expressing embryogenic cell suspension lines

**Figure 3:**
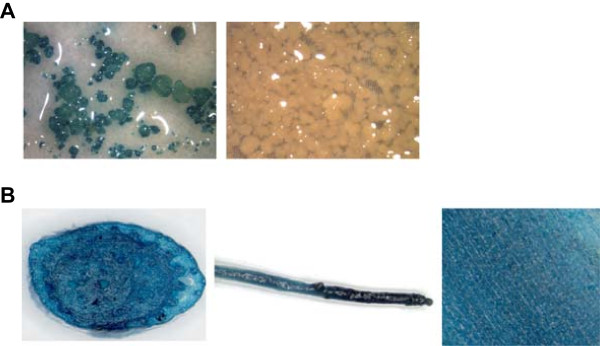
**Histochemical staining of GUS enzyme activity in stable GUS expressing ECS and** ***in vitro*** **plantlets. (A)** pFAJ3000 (p35S::*gusA* ^*INT*^ *)* transformed (left panel) ECS line (i.e. GUS-line) and untransformed (right panel) ECS line; **(B)** pseudostem cross-section (left panel), root (middle panel) and leaf piece (right panel) from a regenerated transgenic *in vitro* plantlet one and a half year after transformation.

In order to mimic a native gene, we generate a stable GUS expressing ECS by *Agrobacterium*-mediated transformation of *gusA*^*INT*^ into banana ECS [39]. Careful selection under a binocular after six weeks of antibiotic selection generated sufficient *gusA*^*INT*^ transformed cell clusters to initiate the creation of GUS expressing cell lines. After nine months, 7 independent cell suspension lines were obtained. β-glucuronidase activity among the cell suspension lines varied more than 30-fold (from 24 ± 1.6 to 852 ± 41 pmol 4-MU h^−1^ μg protein^−1^, data not shown). Following further multiplication of the cell suspension materials, the stability of the GUS activity was assessed every two weeks for six weeks, for three lines selected based on the high level of GUS activity as well as embryogenicity of the suspensions [40]. Flasks of the same line exhibited comparable GUS activity, which was significantly higher in those of cell suspension line no. 11 than of the other two lines (Additional file 2). In addition to strong GUS expression, suspension line no. 11 also exhibited homogenous GUS expression as determined by histochemical GUS staining (Figure 3A) and no contamination was detected. This ECS line, hereafter called the GUS line, was chosen for testing GUS expression throughout *in vitro* development and RNAi-induced gene silencing using the ihpRNA vectors.

#### The GUS expressing banana ECS exhibits constitutive GUS expression throughout *in vitro*development

**Figure 4:**
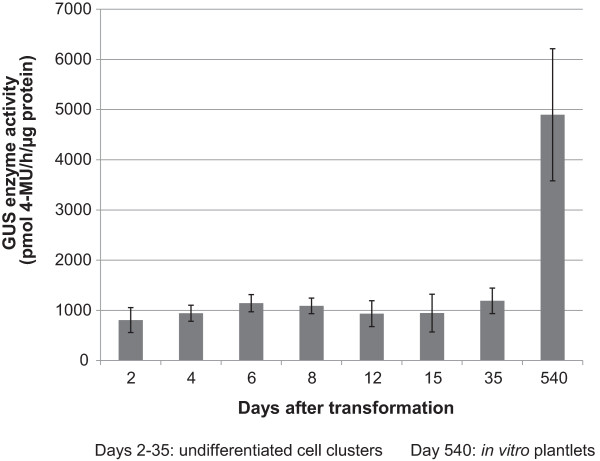
**Analysis of GUS enzyme activity in stable GUS expressing ECS line.** GUS enzyme activity of stable GUS expressing line from 2 to 540 days after transformation with vector pFAJ3000 (p35S::*gusA* ^*INT*^ *)*. From 2 to 35 days cell clusters were proliferating under selective conditions [ZZ medium (pH 5.8) supplemented with 50 mg L^−1^ geneticin, 200 mg L^−1^ timentin], while 540 days after transformation GUS enzyme activity was measured in leaf tissue of *in vitro* plants. Bars represent the mean ± standard deviation (SD). Each mean value was calculated from six independent biological replicates (n = 6) (one replicate of proliferating cell clusters consisted of multiple clusters, while leaf tissue of one plantlet was used per replicate).

To detect the stability of GUS expression from the ECS to the *in vitro* plantlet stage, plants were regenerated from the GUS line. Samples were taken from the control treatment with ZZ medium (non-selective medium) during the transformation with the ihpRNA vectors (see below). Harvesting was done from day 2 after transformation until *in vitro* transgenic plantlets (540 days after transformation) (Figure 4) for fluorometric GUS activity measurement. Regenerated *in vitro* plantlets were obtained approximately 8 months after transformation and subsequently multiplied before sampling. During the stage of undifferentiated cell clusters (from day 2 to 35 after transformation), GUS activity remained very stable (800 – 1200 pmol MU h^−1^ μg protein^−1^), after which it increased 5-fold to approximately 4900 pmol MU h^−1^ μg protein^−1^ in *in vitro* plantlets (Figure 4). A histochemical GUS assay was performed to confirm these results. Stained pseudostem cross-sections, leaf and root pieces of *in vitro* GUS line plantlets exhibited very strong GUS activity (Figure 3B). Histochemical assay results of these transgenic *in vitro* plantlets remained identical when performed up to one year later, i.e. approximately two and a half years after transformation (data not shown). Taken together these results clearly show that GUS expression in the GUS line derived material remained constitutive throughout *in vitro* development and stable within a developmental stage.

#### 299-nt dsRNA causes reduction of GUS enzyme activity and GUS mRNA accumulation

**Figure 5:**
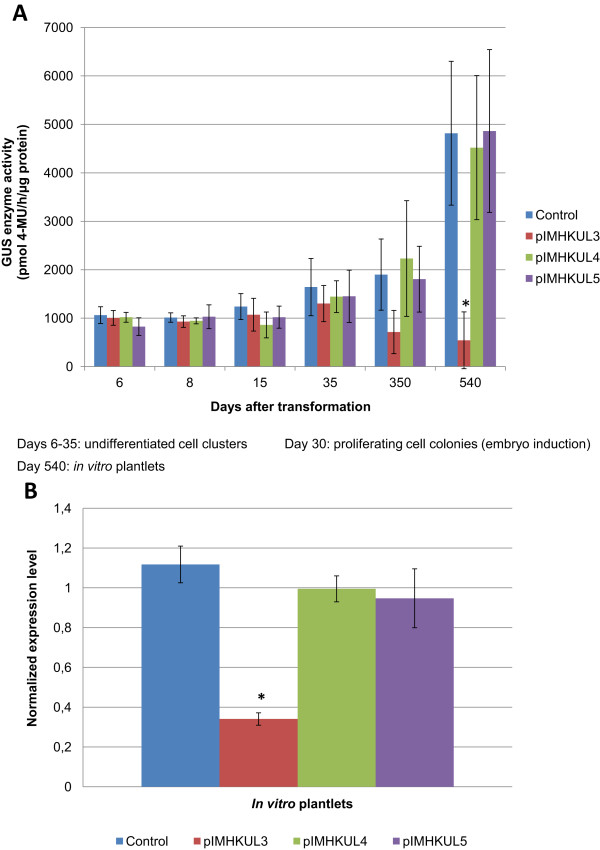
**Analysis of RNAi-mediated silencing in stable GUS expressing banana. (A)** GUS enzyme activity of control (pSTARGATE transformed) and transgenic (ihpRNA vector transformed) lines at different developmental stages after transformation of a stable GUS expressing ECS line (pFAJ3000 transformed). Bars represent the mean ± SD. Mean values of undifferentiated cell clusters (6–35 days after transformation) were calculated from six independent biological replicates (n = 6); those at proliferating cell colonies (embryo induction) stage were measured from 10 independent lines (n = 10); and those at *in vitro* plantlet stage were the average of 12 independent lines per construct (n = 12) with two independent measurements per line. Significant differences with the control were analyzed using one-way ANOVA (*, P < 0.05). **(B)** Expression level of *gusA* ^*INT*^ was determined by RT-qPCR. RNA was isolated from a portion of the leaves used for the GUS enzyme activity assay in **(A)**. Gene expression analyses were performed using two technical replicates per line and the same number of biological replicates or independent lines as in **A**. Bars represented the mean ± SD. *: P < 0.05; Significant differences with the control was analyzed using one-way ANOVA.

**Figure 6:**
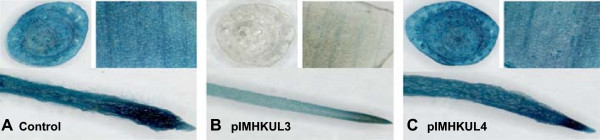
**Histochemical staining of GUS enzyme activity in transgenic banana** ***in vitro*** **plantlets harbouring an ihpRNA vector.** GUS staining of a pseudostem cross-section, leaf piece and root from *in vitro* plantlets regenerated after transformation of a stable GUS expressing (pFAJ3000 transformed) ECS with the empty control cloning vector pSTARGATE **(A)**, the ihpRNA vector pIMHKUL3 **(B)** or ihpRNA vector pIMHKUL4 **(C)**. Samples were excised two and a half years after transformation.

We next investigated the silencing effect of the ihpRNA vectors on stable GUS expression. The ihpRNA vectors pIMHKUL3, pIMHKUL4 and pIMHKUL5 were individually transformed into the GUS line ECS via *Agrobacterium tumefaciens* [39] and transgenic lines were produced under hygromycin selection. The pSTARGATE cloning vector, containing no GUS sequence, was also transferred as positive control. For each vector more than 100 independent transgenic lines were regenerated, of which the 20 most vigorously growing were selected for PCR screening to confirm the presence of the inverted repeat (IR) cassettes. Twelve confirmed independent lines per vector were selected for further study (data not shown). At an early developmental stage in undifferentiated cell clusters (day 6–35 after transformation), no reduction of GUS activity was detected irrespective of the *gusA* target sequence used for silencing (Figure 5A). At proliferating cell colony (embryo induction) stage, a trend of reduction in GUS activity, though not statistically significant, occurred in pIMHKUL3 lines compared to the control lines, while GUS activity in pIMHKUL4 and pIMHKUL5 lines remained comparable to that of the control lines. 540 days after transformation, leaf tissue of the regenerated *in vitro* plantlets was also tested and GUS activity reached approximately 2.5-fold higher than the level at cell colony stage in the control lines (approx. 2000 vs. 5000 pmol MU h^−1^ μg protein^−1^, respectively; Figure 5A), which is in agreement with the GUS activity measured earlier in the untransformed GUS line derived *in vitro* control plantlets (Figure 4). All pIMHKUL3 lines tested exhibited a reduced GUS activity compared to the pSTARGATE control at the *in vitro* plantlet stage, albeit it to a variable degree. On average this down-regulation amounted up to 9-fold, whereas GUS activity was not affected in the pIMHKUL4 and pIMHKUL5 lines (Figure 5A). To confirm the expected down-regulation of *gusA* transcripts in pIMHKUL3 transgenic *in vitro* plantlets, real-time quantitative PCR (RT-qPCR) analysis with *gusA* specific primers (Table 1) was performed. RNA was isolated from a portion of the *in vitro* leaf material used for the GUS enzyme activity assay. All (100%) pIMHKUL3 lines showed a reduction of *gusA* mRNA accumulation compared to the pSTARGATE control resulting in an average 4.5-fold down-regulation (Figure 5B). In contrast, the pIMHKUL4 and pIMHKUL5 transformed lines did not exhibit lower *gusA* transcript accumulation than the control lines. These RT-qPCR results were consistent with the GUS activity data and demonstrated that siRNA-guided *gusA* mRNA degradation occurred in pIMHKUL3 lines but not in pIMHKUL4 and pIMHKUL5 ones.Finally, GUS histochemical analyses revealed that the pseudostem, leaf and root tissues of the majority of the pIMHKUL3 transgenic plantlets exhibited strongly reduced GUS staining compared to the pSTARGATE control, whereas pIMHKUL4 lines stained equally blue (Figure 6). Tissues of pIMHKUL5 transformed plantlets stained similar as the control as well (data not shown).

**Table 1:** **List of primer sequences used in this study**

Primers	Sequence 5′-3′
GUS shl	CACCCTGCTGTCGGCTTTAACCTC
GUS shr	GTGAGCGTCGCAGAACATTA
GUS-qPCR-F1	TGTGGAGTATTGCCAACGAA
GUS-qPCR-R1	GAGCGTCGCAGAACATTACA
KUL3-N- F	CTGCTGTCGGCTTTCAGCTG
KUL3-T7-N-R	TAATACGACTCACTATAGGGGTGAGCGTCGCAGAACATTA
EF1-F2	CGGAGCGTGAAAGAGGAAT
EF1-R2	ACCAGCTTCAAAACCACCAG
L2-F2	AGGGTTCATAGCCACACCAC
L2-R2	CCGAACTGAGAAGCCCCTAC
tub-F1	TGTTGCATCCTGGTACTGCT
tub-R1	GGCTTTCTTGCACTGGTACAC
Act-F4	GAGAAGATACAGTGTCTGGA
Act-R4	ATTACCATCGAAATATTAAAAG
UbiL4	GTCGATGCTCACCCTGTTGT
NosT3-2	ACCGGCAACAGGATTCAA
35SL	AATATCGGGAAACCTCCTCG
35SR	AAGGATAGTGGGATTGTGCG
GUS-HPT-GT3R	ACGCTGATCAATTCCACAG
GUS-SAGE-R	CCCTGCTGCGGTTTTTCACCGAAGTT

Overall, the results of the fluorometric and histochemical GUS assays, as well as the RT-qPCR analysis of *gusA* mRNA accumulation indicated that RNAi was functional and GUS gene expression was stably suppressed in the pIMHKUL3 transformed banana lines.

#### GUS expression levels relate well with siRNA expression

**Figure 7:**
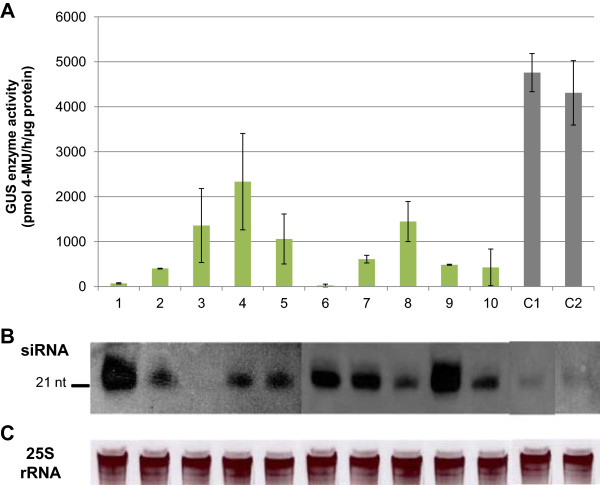
**Relation between GUS and siRNA expression in transgenic** ***in vitro*** **plantlets containing the pIMHKUL3 vector. (A)** GUS enzyme activity in leaves of 10 independent pIMHKUL3 transgenic lines (1–10) and of 2 pSTARGATE control lines (C1, C2) regenerated after transformation of a stable GUS expressing (pFAJ3000 transformed) ECS. Bars represented the mean ± SD. Mean values were calculated from two independent measurements per line. **(B)** Northern blot detection of small RNA of ~21 nt using a *gusA* RNA probe. RNA was isolated from a portion of the leaves used for the GUS enzyme activity assay in **(A)**. **(C)** 25S rRNA from the Northern gel blot from **(B)** was used as loading control.

The above results demonstrated PTGS of the *gusA* gene in pIMHKUL3 transformed *in vitro* plantlets. We next performed Northern blotting to detect the presence of GUS-specific siRNAs produced by the 299-nt dsRNA transgene in these lines, being the hallmark of PTGS [41], and if so, whether their expression inversely related with GUS expression. Ten independent pIMHKUL3 lines with the strongest *gusA* gene silencing and two pSTARGATE control lines were selected. As shown in Figure 7, using a 299-nt RNA DIG-labeled GUS probe, GUS-specific siRNAs of about 21-nt were detected in all samples except sample 3. Small RNAs of this size were shown to activate homology-dependent degradation of target RNAs [36, 42, 43]. Moreover, the abundance of this specific siRNA was inversely related with GUS activity except in samples 2 and 3 (Figure 7B and A, respectively), indicating that siRNA-guided degradation of target *gusA* mRNA was taking place in the former lines. In addition, siRNAs were also detected in control lines even though their expression was extremely low (Figure 7B).

In conclusion, the results demonstrated effective and stable *gusA* gene silencing through the expression of *gusA* gene-specific siRNAs in stable GUS expressing banana.

## Discussion

In many organisms, RNAi or PTGS is a gene silencing approach resulting in specific degradation of endogenous RNA in the presence of homologous dsRNA either locally injected or transcribed from an IR transgene [44]. In the beginning, most studies to assess RNAi-mediated gene silencing in plants have been performed on transgenic plants expressing an active or silenced reporter gene [36, 37, 41, 45–47]. In this study, we have demonstrated that RNAi-mediated gene silencing can efficiently down-regulate stable GUS expression in banana. Besides the advantage of visual monitoring of the silencing effect by histochemical GUS staining, we also could quantitatively measure it at the mRNA as well as enzyme activity level. The results significantly add to the functional genomics research in banana, which aims to exploit agronomically valuable genes for genetic improvement of this important crop.

The design of the ihpRNA vectors is a critical factor of a gene silencing study. It was reported that the synthetic siRNAs of 21-22-nt have been successfully used for inducing strong and specific RNAi gene silencing [36, 43]. In addition, RNAi-induced gene silencing was also shown to be stable and efficient using longer homologous sequences, i.e. around 500-nt, 303-nt, 745-nt and 474-nt to suppress the *PDS* and *EIN2* genes in wheat [19], the *FaCHS* gene in strawberry fruit [48], the *COI1* gene in rice [28] and the *Rar1* gene in potato (*Solanum tuberosum*) [49], respectively. Furthermore, in plants, ihpRNA constructs were shown to give higher gene silencing efficiency than intron-free hpRNA ones since the intron spacer makes the interaction of the two arms of the hairpin more probable [46, 50, 51]. Therefore, we used an ihpRNA vector backbone to construct our silencing vectors, containing target sequences of various lengths (19-nt, 299-nt, and the 26-nt SuperSAGE tag).

Previously, RNAi-mediated transient gene silencing assay using a reporter gene was successfully exploited in plants by co-infiltrating green fluorescent protein (GFP) and constructs yielding dsGFP in tobacco leaves [41, 45]. Results of these authors demonstrated that dsGFP repressed GFP expression via PTGS. More recently, RNAi-mediated transient gene silencing was applied to suppress expression of endogenous genes in various crop plants such as potato [49], rice [31], grapevine (*Vitis vinifera* L.) [52] and other plant species including *Stevia rebaudiana* [53] and *Betula platyphylla Suk* [54], thereby, becoming a powerful approach for the functional assay for a large number of candidate genes. In this study, RNAi-mediated transient gene silencing was also obtained via co-transformation of GUS and ihpRNA GUS constructs in banana ECS. At day 2 and 4 after co-transformation, very low GUS expression in all lines caused insignificant differences in GUS activity between the control and any of the co-transformed ihpRNA vectors. GUS expression was highly elevated in all lines in the following days. Reduction of GUS activity was detected at days 6 and 8 after co-transformation in pIMHKUL3 and pIMHKUL5 co-transformed samples (Figure 2). These results indicate that transient gene silencing can be an effective approach for quick assessment of the functionality of endogenous genes in banana as well. Besides, due to the polyploidy and heterozygous nature of the banana genome, designing dsRNA constructs needs to be adapted so that the dsRNA can target multigene families or multi-copies of a gene for complete and efficient silencing.

A key component of this study is the successful generation of a stable GUS expressing banana ECS line (i.e. the GUS line), which allowed us to study the silencing of a stably integrated gene in banana. Previously, it was reported that in *Santalum album* L. stability of GUS expression in *gusA*-transformed ECS was successfully obtained [55]. In this study, multiple GUS expressing cell suspension lines with stable, albeit variable, levels of expression were generated demonstrating the reproducibility and efficiency of the approach. The selected GUS line also showed constitutive GUS expression during development with a strong increase in *in vitro* plants where it remained stable during several *in vitro* subcultures over a period of one year. Hence, any silencing effect induced by the ihpRNA vectors was caused by siRNA-mediated *gusA* mRNA degradation rather than the instability of GUS expression. In addition, to the best of our knowledge, this study is the first demonstrating successful generation of transgenic ECS for second transformation with (an)other construct(s) in banana. This finding adds another value to functional genomics study of interactions of endogenous genes in banana.

RNAi-induced gene silencing research using a stable GUS expressing system has been successfully conducted in different model plants [36, 37]. However, to the best of our knowledge this study is the first that uses a stably integrated *gusA* gene to investigate gene silencing in banana. The ihpRNA vectors were transformed individually into the GUS line ECS, and stable silencing of GUS expression was investigated in transgenic lines from day 6 up to one and half years after transformation. Fluorometric GUS assay results showed that reduction of GUS activity was undetectable in ihpRNA vector-transformed undifferentiated cell clusters in comparison to control. This can be due to the fact that each transgenic replicate harvested was a mixture of ihpRNA vector transformed and non-transformed GUS line ECS, which all showed very strong GUS expression. Therefore, siRNA-mediated silencing of GUS might occur in transformed suspension cells but this signal was overwhelmed by GUS expression in non-transformed ones. The transgenic pIMHKUL3 lines exhibited low GUS activity in cell colonies as well as in *in vitro* plantlets, which proved that PTGS functioned stably and constantly and that *GUS* mRNA was continuously degraded by pIMHKUL3-induced siRNAs. Albeit to a variable degree, 100% of the investigated pIMHKUL3 transgenic *in vitro* lines showed silencing of GUS expression. This is higher than the results obtained in tobacco, rice and reporter gene silencing in other silencing systems mentioned above [36, 37, 56], thereby proving the efficiency of the RNAi approach in banana. Consistent with the observed GUS activity in transgenic *in vitro* plantlets, *gusA* transcript accumulation in all the investigated transgenic pIMHKUL3 lines was significantly reduced, whereas it did not change in the transgenic pIMHKUL4 and pIMHKUL5 lines compared to control. These results of GUS activity and RT-qPCR were confirmed by GUS histochemical assay. Reduction of *gusA* transcript accumulation and GUS activity in transgenic pIMHKUL3 *in vitro* plantlets was proven to be mediated by siRNA produced from 301 bp RNA duplex. All these observations demonstrate that RNAi-mediated gene silencing machineries efficiently and stably function in banana, providing a reliable basis for application of silencing of native banana genes in the future.

It was suggested that over-expression of transgene RNA above a putative threshold triggers degradation of the transgene RNA [57]. In our study, the stable GUS expressing banana control plants produced high amounts of *gusA* RNA as shown by the GUS enzyme activity in these plants (Figure 4) as well as the mRNA expression level in the ihpRNA empty vector pSTARGATE transformed plantlets (Figure 5B). This might activate plant PTGS to suppress the *gusA* RNA which could explain the relatively low siRNA expression in a few pSTARGATE transgenic plantlets via Northern blot detection (Figure 7B).

The ihpRNA vectors pIMHKUL4 and pIMHKUL5 yielding 28 bp and 21 bp RNA duplexes, respectively, did not cause any silencing effect although the latter successfully suppressed GUS expression in tobacco [36]. It might be explained by the fact that in Lu et al. [36] the size of intron spacer between the sense and antisense target sequence was only 9 nt in length, whereas in our ihpRNA constructs the intron spacer of 1183 nt was much larger than the *gusA* target sequences in pIMHKUL4 and pIMHKUL5 (1183 nt vs. 26 nt and 19 nt, respectively). This might prevent the gene silencing pathway in banana to recognize these dsRNAs to trigger PTGS. Consequently, no siRNA is produced to induce GUS silencing.

## Conclusions

Transgenic banana have been generated in the past years in order to increase resistance against devastating banana diseases such as Fusarium wilt [58], bunchy top [59]. Besides disease resistance, in the recent years, fortification in banana has also raised interest since it can help to solve the problem of malnutrition, especially in poor countries. Iron-fortified bananas were generated to reduce iron deficiency [60] while vitamin A-fortified bananas have been reported to be introduced in the near future by scientists in Queensland University of Technology (Brisbane, Australia). Nevertheless, most of transgenic bananas are generated by modifying with genes from other species. The future duty is to initiate transgenic banana plants using banana endogenous genes. For this purpose, RNAi-mediated gene silencing will play an important role in defining functions of the banana endogenous genes.

Our study experimentally demonstrated RNAi-mediated gene silencing in banana using the *gusA* reporter gene as a model system. The early screening of ihpRNA vectors by a transient expression assay revealed the potential of *gusA* transgene silencing. RNAi-induced silencing of stable GUS expression was proved feasible in banana and the silencing effect at mRNA level was tightly related with that at GUS enzyme activity level. This result, together with successful generation of transgenic ECS warrants further silencing studies of candidate agronomically important native genes and their interactions as well for functional characterization in this important crop. Finally, the use of a reporter gene as a model system to optimize gene silencing is an alternative test system to one based on a native gene due to its precise quantification at both mRNA and enzyme activity level.

## Methods

### Plant material

Embryogenic cell suspensions (ECS) of the banana cultivar ‘Williams’ (*Musa* spp. AAA group) were maintained in liquid ZZ medium containing 5 μM 2,4-D and 1 μM zeatin [61]. The suspension was initiated from *in vitro* multiple meristem cultures [40]. Cells were maintained on a rotary shaker (70 rpm) at 26 ± 2°C under continuous light of 50 μE m^−2^ s^−1^ and subcultured every 2 weeks as described [40].

### Generation of a stable GUS expressing banana embryogenic cell suspension

Plasmid pFAJ3000 containing a *gusA-intron* (*gusA*^*INT*^) gene driven by the CaMV 35S promoter and a neomycin phosphotransferase (*npt*II) selectable marker gene cassette [62] was transferred to *Agrobacterium tumefaciens* strain EHA101 [63].

Successfully transformed bacteria were cultured at 28°C for 48 h on solid yeast-mannitol medium (0.4 g L^−1^ yeast extract, 10 g L^−1^ mannitol, 0.5 g L^−1^ K_2_HPO_4_.3H_2_0, 0.2 g L^−1^ MgSO_4_.7H_2_0, 0.1 g L^−1^ NaCl, pH 7.0) containing 300 mg L^−1^ streptomycin (Sm^300^) and 100 mg L^−1^ spectinomycin (Sp^100^). Single colonies were picked and shaken in selective liquid yeast-peptone medium (10 g L^−1^ yeast extract, 10 g L^−1^ peptone, 5 g L^−1^ NaCl) at 28°C and 210 rpm for 30 h. *Agrobacterium*-mediated transformation of banana ‘Williams’ ECS cultures was performed as reported [39]. Samples mixed with only ZZ medium (half strength MS medium supplemented with 5 μM 2,4-D and 1 μM zeatin, pH 5.6) during the 6 h infection period were included as negative untransformed control.

Following 7 days co-cultivation, plastic Petri dishes (5 cm diameter) containing transformed ECS spread on a sterile 50 μm polyester mesh and placed on selective (50 mg L^−1^ geneticin) ZZ medium (pH 5.8) supplemented with 200 mg L^−1^ timentin were incubated in the dark for approximately 6 weeks at 25 ± 2°C. Untransformed control samples were maintained on non-selective ZZ medium. A bi-monthly subculture regime to fresh selective ZZ medium in the case of transformed ECS and non-selective ZZ medium for untransformed control was followed. Under a binocular, transgenic early stage transparent proembryos (<1 mm in size) and small groups of undifferentiated control cell clusters were collected and transferred to a 25 mL Erlenmeyer flask containing 10 mL liquid non-selective ZZ medium. Approximately 50 transgenic proembryos or groups of control cell clusters were cultured per flask under standard conditions (i.e. on a rotary shaker at 70 rpm and 26 ± 2°C) with two subcultures per week. At each subculture during the establishment period, all flasks were checked for contamination and embryogenicity under an inverse microscope. Dead as well as floating cell material was carefully removed using a plastic pipet. After 14–15 weeks the suspensions with the most embryogenic cell clusters were transferred to a 50 mL Erlenmeyer flask containing 20 mL non-selective ZZ medium for 1–2 weeks and maintained under the same standard conditions subculture regime. Subsequently, culturing took place in a 100 mL Erlenmeyer flask (40 mL non-selective ZZ medium) with a weekly subculture for another 4 weeks. Finally, ECS lines were maintained in 250 mL Erlenmeyer flasks (90 mL non-selective ZZ medium) under standard conditions and subcultured every 2 weeks.

In order to eliminate possible remaining agrobacteria, four different treatments starting from the collection of proembryos and cell clusters were compared during the initiation of ECS lines: (i) ZZ medium supplemented with 200 mg L^−1^ timentin, (ii) ZZ medium supplemented with 200 mg L^−1^ timentin during the first 3 months only, (iii) ZZ medium supplemented with 200 mg L^−1^ timentin for 3 months and 100 mg L^−1^ subsequently, (iv) ZZ medium only. The absence of agrobacteria was verified after 5 and 7 weeks in liquid ZZ medium by plating small samples of suspension cells on non-selective as well as selective (Sm^300^ + Sp^100^) Bact (5 g L^−1^ yeast extract, 10 g L^−1^ sucrose, and 23 g L^−1^ nutrient agar, pH 7.0) and YM (0.1 g L^−1^ NaCl, 0.2 g L^−1^ MgSO4 · 7H2O, 0.5 g L^−1^ K_2_HPO_4_ · 3H_2_O, 0.4 g L^−1^ yeast extract, 10 g L^−1^ mannitol, and 13 g L^−1^ bacto agar, pH 7.0) medium. Petri-dishes were incubated at 28°C. Lines showing bacterial growth under selective conditions were discarded. Later in the procedure samples were plated on Bact as well RD1 (half strength MS medium, 100 mg L^−1^ myo-inositol) media and incubated at 28°C and 37°C to reveal possible contaminations. At regular time intervals throughout the establishment of the *gusA*^*INT*^ transformed suspension lines, samples were checked for homogeneous GUS expression by histochemical staining (see below). Samples that did not stain completely blue were discarded. Finally, one of the retained ECS line staining dark blue was selected for transformation with intron-spliced hairpin (ihpRNA) constructs to assess RNAi-induced silencing.

### Preparation of RNAi vectors

Three ihpRNA vectors pIMHKUL3, pIMHKUL4 and pIMHKUL5 targeting different sites of the *gusA* mRNA sequence were created using the Gateway-enabled backbone vector pSTARGATE (supplied by CSIRO Plant Industry, Australia) [64]. For the pIMHKUL3 vector, a 299-nt sequence targeting the sequence 1273–1572 downstream of the A^+1^TG translation start towards the 3′end of the *gusA*^*INT*^ coding sequence was PCR-amplified from the pFAJ3000 vector using the forward primer gus shl and the reverse primer gus shr (Table 1). The PCR product was cloned into pENTR™/D-TOPO® (Life Technologies™, Invitrogen™, Ghent, Belgium) following the manufacturer’s instructions. After confirming the accuracy of the insert, a Gateway Recombinase reaction was performed according to the manufacturer’s instruction to transfer this 299 nt insert into pSTARGATE to form ihpRNA vector pIMHKUL3.

A 26-nt sequence targeting the sequence 40–66 upstream of the *gusA-intron* stop codon TGA coinciding with the expected 26 bp tag that would be retrieved for the *gusA*^*INT*^ gene by SuperSAGE analysis [38] was used to construct the pIMHKUL4 vector. The SuperSAGE oligo (5′-AACTTCGGTGAAAAACCGCAGCAGGG-3′) was synthesized and cloned into pSTARGATE as described above to create ihpRNA vector pIMHKUL4.

To form ihpRNA vector pIMHKUL5, a 19-nt sequence (5′-CTGTGGAATTGATCAGCGT-3′) targeting the sequence 81–99 downstream of the *gusA-intron* start codon ATG retrieved from Lu et al. [36] was synthesized and cloned into pSTARGATE as described above (Additional file 1). The pIMHKUL3, pIMHKUL4 and pIMHKUL5 will yield the TT-overhang RNA duplexes of 301-, 28- and 21- bp, respectively. Following sequence confirmation, the ihpRNA vectors as well as pSTARGATE were transferred to *A. tumefaciens* strain EHA105 [65].

### Generation of RNAi lines

For transient GUS silencing the ihpRNA constructs were individually introduced into ECS together with the *gusA*^*INT*^ containing vector pFAJ3000 by *Agrobacterium*-mediated transformation as reported previously [39]. Each ECS sample was infected with a 1 mL mixture of agrobacteria consisting of 500 μL of each strain. Co-transformation of pSTARGATE and pFAJ3000 was also performed as control. The co-cultivated cells were harvested from 2 to 8 days after transformation with intervals of 2 days and GUS activity was measured fluorometrically.

For silencing of stable GUS expression, the ihpRNA vectors and pSTARGATE control vector were individually transferred into the stable GUS expressing ECS by *Agrobacterium*-mediated transformation. Subsequent selection and regeneration of transgenic lines were performed according to Pérez Hernández et al. [39].

### Total DNA isolation and polymerase chain reaction (PCR)

Total banana DNA was isolated from *in vitro* leaf tissue as previously described [7]. PCR was performed in 0.2 mL microfuge tubes in a Mastercycler Gradient™ cycler (Eppendorf, Hamburg, Germany) in a final volume of 20 μL. Reactions were programmed to an initial denaturation for 5 min at 95°C followed by 35 cycles of 95°C for 20 s, 55°C for 20 s, and 72°C for 1 min and a final elongation step for 7 min at 72°C. PCR products were observed under UV light after 0.8% (w/v) agarose gel electrophoresis.

### Reverse transcriptase quantitative PCR analysis of *gusA*^*INT*^gene expression

*In vitro* plant leaf tissue was harvested, immediately frozen in liquid nitrogen and stored at −80°C until further use. Total RNA was isolated according to Aljanabi et al. [66] with modifications. Leaf tissue was ground in liquid nitrogen and homogenized in 1.5 mL extraction buffer [400 mM NaCl, 10 mM Tris–HCl pH 8.0, 2 mM Na_2_-EDTA, 2% (m/v) polyvinyl pyrrolidone MW 40,000, 0.01% (v/v) β-mercaptoethanol, 2% (m/v) sodium dodecyl sulphate] for 30 seconds followed by incubation at 55°C for 1 h and phenol/chloroform and chloroform extractions. After precipitation in 6 M LiCl, RNA was pelleted and dissolved in 100 μL water. The extracted RNA was treated with RNase-free Ambion® DNaseI (Life Technologies™, AB Applied Biosystems™, Ghent, Belgium), which was subsequently removed during a phenol-chloroform/ethanol purification step. Using the Nanodrop ND-1000™ spectrophotometer (Nanodrop Technologies, Wilmington, DE, USA), the quantity and quality of total RNA were measured, while the absence of gDNA was verified as described [67]. Subsequently, 1 μg of each DNA-free RNA sample was reverse-transcribed to cDNA using the RevertAid H Minus First Strand cDNA Synthesis Kit (Fermentas, St- Leon Rot, Germany) according to the manufacturer’s instructions.

Real-time PCR using the Corbett Rotor-Gene 3000 (Qiagen, Hilden, Germany) was essentially performed as described [67]. Briefly, the total reaction volume of 25 μL contained 1 X ABsolute™ qPCR SYBR® Green Mix (Thermo Scientific, Epsom, UK), 125 ng λ-DNA (Roche Diagnostics, Vilvoorde, Belgium), 100 nM of each specific sense and anti-sense primers (Table 1), and 2 μL of a 12 X diluted template cDNA. Cycling conditions encompassed a polymerase activation at 95°C for 15 min and then 45 cycles of 95°C for 15 s, 52-62°C for 20 s, and 72°C for 20 s with a final elongation at 79-86°C for 15 s. At the end of each run a melting curve was generated from 55 to 95°C to verify the specificity of the amplicon. A standard curve of six serial 4-fold dilution of a *gusA* RT-qPCR product of 135 bp length [using GUS-qPCR-F1/R1 primers (Table 1) and the RT-qPCR program described above with annealing temperature at 58°C for 20 s and a final elongation at 86°C], a no-template water control, and the cDNA samples each with two technical replicates were always run concurrently in each assay. Reference genes *TUB* and *EF1* were selected for RT-qPCR using geNorm 3.4 following instructions from Podevin et al. [67]. To calculate the normalized expression level of *GUS* mRNA, the quantity of GUS (Q_GUS_) and two reference genes (Q_ref1_ and Q_ref2_) were first generated from the Ct mean value (average Ct of 2 replicates). Quantities of the two reference genes were then used to calculate the normalization factor [NF = GEOMEAN (Qref_1_; Qref_2_)]. Finally, the GUS normalized expression level was calculated as Q_GUS_/NF. Statistical analysis for significant difference of normalized expression level was performed using one-way ANOVA.

### GUS activity assays

#### Histochemical GUS assay

Plant materials were stained with X-GLUC (1 mg/mL) in 100 mM Tris–HCl (pH 8.0) containing 10 mM EDTA, 0.5 mM potassium ferrocyanide, 0.5 mM potassium ferricyanide, 1% ascorbic acid and 0.2% CHAPS at 37°C overnight. After staining, plant tissues were decolourized by ethanol and photographed under a WILD M3 (Wild Heerbrugg, Switzerland) binocular using the Microscopica software.

#### Fluorometric GUS assay

Plant materials were ground in liquid nitrogen and homogenized in 500 μL extraction buffer [20% (v/v) methanol, 50 mM phosphate buffer (pH 7.0), 10 mM EDTA, 0.07% (v/v) β-mercaptoethanol, 0.1% (m/v) sodium lauryl sarcosine, 0.1% (v/v) Triton X-100, 2% (m/v) polyvinyl pyrrolidone MW 10,000] followed by centrifugation. The supernatant was stored in aliquots at −80°C for further measurements.

Protein concentration was measured following the Bradford assay [68] using ‘Coomassie brilliant blue G250’ protein staining solution (0.08% Coomassie brilliant blue G250, 1.6% ortho-phosphoric acid, 8% ammonium sulphate, 20% methanol). Following preparation of a 96-well transparent plate in which each well contained 20 μL of protein extract and 200 μL of protein staining solution, absorbance was measured at 595 nm. Each sample was measured in duplicate with several dilutions per replicate. A BSA dilution series (31.25 to 1000 ng mL^−1^) was included in each plate to establish a standard curve. The GUS activity was analyzed according to Jefferson et al. [69]. The enzymatic reaction was performed with 50 μL protein extract in 200 μL of 1 mM MUG assay buffer [4 mg MUG (Duchefa) dissolved in 10 mL extraction buffer] at 37°C for 1 h. MU produced by the enzymatic reaction was measured fluorescently (excitation wavelength 363 nm and emission 474 nm) in 96-well black plates in which each well contained 10 μL of reaction and 190 μL of stop buffer (0.2 M Na_2_CO_3_) to stop the reaction. Each sample was measured in duplicate with at least two dilutions per replicate. A MU (Duchefa) dilution series (50 to 10,000 nM) was included in each measurement to generate a standard curve. Both protein concentration and fluorescence were measured using a Synergy MX Monochromator-Based Multi-Mode Microplate Reader (BioTek).

### Northern blot analysis of siRNA expression

Total RNA was isolated as described above with the following modifications to recover small RNAs: after incubating the mixture of leaf tissue in extraction buffer at 55°C for 15 min, total RNA was isolated by acidified phenol:chloroform (1:1) (Sigma), followed by isopropanol precipitation. 5 μg of the extracted, DNA-free RNA was denatured at 95°C for 5 min and separated in a 15% polyacrylamide/7 M urea gel (Sigma) at 180 V in 0.5 X TBE buffer using a Protean II apparatus (BioRad) until the bromophenol blue dye reached the bottom of the gel. The separated RNA was transferred onto a positively charged nylon membrane (Roche Diagnostics, Vilvoorde, Belgium) using a Trans-Blot SD Semi-Dry Electrophoretic Transfer Cell (BioRad Laboratories, Nazareth Eke, Belgium). The membrane was auto-crosslinked at 120,000 μJ in a Stratalinker 1800 (Agilent Technologies, Diegem, Belgium), prehybridized in DIG Easy Hyb hybridization solution (Roche Diagnostics, Vilvoorde, Belgium) at 50°C for 30 min and hybridized with a DIG-labeled RNA probe spanning 300 bp of the *gusA*^*INT*^ coding sequence (sequence 1273–1573) at 50°C for 16 h in a hybridization oven. DIG-labeled RNA probe preparation was done as follows. PCR amplification with primers KUL3-N-F and KUL3-T7-N-R (Table 1) was performed on template pFAJ3000 plasmid DNA (5 ng per reaction). After cleaning by a phenol-chloroform/ethanol purification step, 1 μg of PCR product was used for RNA probe labeling by T7 RNA polymerase according to the Instruction Manual of the DIG Northern Starter Kit (Roche) (https://cssportal.roche.com/LFR_PublicDocs/ras/12039672910_en_07.pdf). Post-hybridization washes and immuno-chemiluminescent detection of the bound probe were also performed following the Instruction Manual of the DIG Northern Starter Kit (Roche). The membrane was subsequently exposed to X-ray film (Thermo Scientific, Massachusetts, USA) for 5 min and developed to visualize the hybridization signals.

## Electronic supplementary material

Additional file 1: **Schematic representation of positions of the 299-nt, 26-nt and 19-nt sequences in** ***gusA*** ^***INT***^ **gene.** These sequences were cloned in the pSTARGATE vector to form the ihpRNA constructs pIMHKUL3, pIMHKUL4 and pIMHKUL5, respectively. (PDF 48 KB)

Additional file 2: **Analysis of GUS enzyme activity of different GUS expressing embryogenic cell suspension lines.** GUS enzyme activity of different GUS expressing embryogenic cell suspension (ECS) lines contained in various flasks was detected at 2, 4, and 6 weeks after subculture. Bars represent the mean (±SD) of the 3 different time points. ECS line 11 was chosen for testing the different ihpRNA vectors. (PDF 7 KB)

## Abbreviations

ECS: Embryogenic cell suspension
CaMV: Cauliflower mosaic virus
BSA: Bovine serum albumin
MUG: 4-methyl umbelliferyl-β-D-glucuronide
MU: 4-methylumbelliferone
MS medium: Murashige and Skoog medium
CHAPS: 3-[(3-cholamidopropyl)dimethylammonio]-2-hydroxy-1-propanesulfonate
DIG: Digoxigenin.
