# Supplementary figures and images for: Assessment of RNAi-induced silencing in banana (Musa spp.)
Source: BMC Res Notes. 2014 Sep 18;7:655. doi: 10.1186/1756-0500-7-655 (PMC4177175; doi:10.1186/1756-0500-7-655)

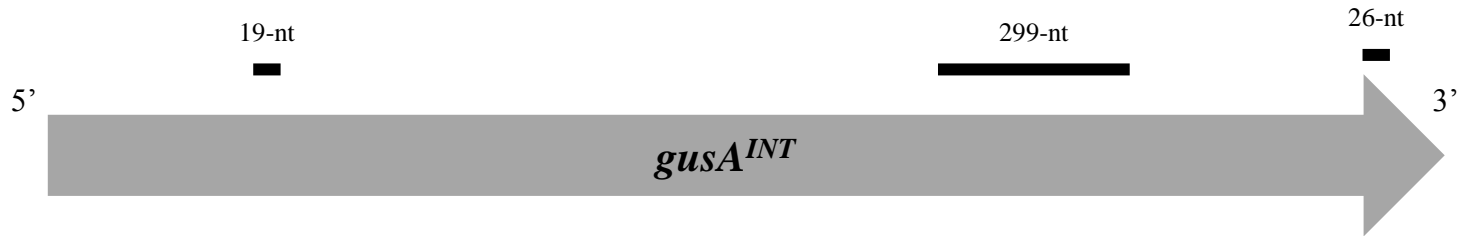

**Additional file 1**

Supplement: Supplementary file 1 — Additional file 1: Schematic representation of positions of the 299-nt, 26-nt and 19-nt sequences in gusA INT gene. These sequences were cloned in the pSTARGATE vector to form the ihpRNA constructs pIMHKUL3, pIMHKUL4 and pIMHKUL5, respectively. (PDF 48 KB) [file 13104_2014_3181_MOESM1_ESM.pdf]

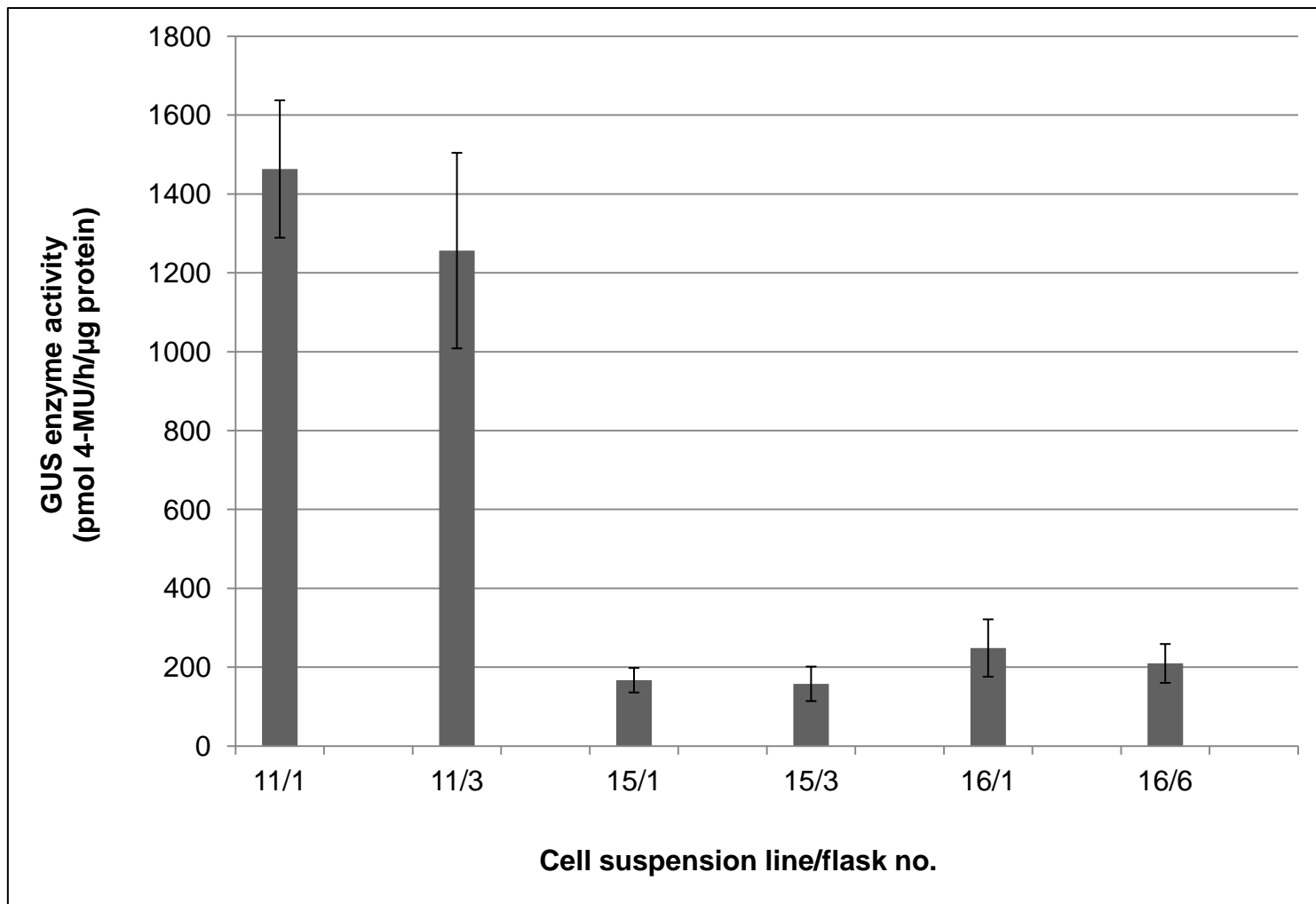

**Additional file 2**

Supplement: Supplementary file 2 — Additional file 2: Analysis of GUS enzyme activity of different GUS expressing embryogenic cell suspension lines. GUS enzyme activity of different GUS expressing embryogenic cell suspension (ECS) lines contained in various flasks was detected at 2, 4, and 6 weeks after subculture. Bars represent the mean (±SD) of the 3 different time points. ECS line 11 was chosen for testing the different ihpRNA vectors. (PDF 7 KB) [file 13104_2014_3181_MOESM2_ESM.pdf]
